# Imaging performance of a full-ring prototype PET-MRI system based on four-layer DOI-PET detectors integrated with a RF coil

**DOI:** 10.1186/2197-7364-2-S1-A18

**Published:** 2015-05-18

**Authors:** Fumihiko Nishikido, Hideaki Tashima, Mikio Suga, Naoko Inadama, Yoshida Eiji, Takayuki Obata, Taiga Yamaya

**Affiliations:** National Institute of Radiological Sciences, Chiba, Japan; Chiba University, Chiba, Japan

We are developing a PET system integrated with a birdcage RF-coil for PET-MRI in order to realize both high sensitivity and high spatial resolution of the PET image by using the 4-layered depth-of-interaction (DOI) PET detector. We constructed a full-ring prototype system and evaluated performances, especially imaging performance, of the prototype system in simultaneous measurement. The prototype system consists of eight four-layer DOI-PET detectors and a prototype birdcage RF-coil developed for the proposed system. The PET detectors consist of six monolithic multi-pixel photon counter array (S11064-050P), a readout circuit, fourlayer DOI scintillator arrays and a shielding box made of 35 μm thick copper foil. The crystal array consists of 2.0 mm x 2.0 mm x 5.0 mm LYSO crystals arranged in 38 x 6 x 4 layer. The RF-coil has eight coil elements and the eight PET detectors are positioned at each element gap. The diameter of the RF-coil elements is 261 mm. We conducted performance tests of the prototype system with a 3.0 T MRI (MAGNETOM Verio). Only the PET detectors, the RF-coil and the cables were in an MRI room during measurements. A data acquisition system and power supplies for the MPPCs and preamplifiers were outside the MRI room and connected to all the detectors through a penetration panel. As a result, the spatial resolutions of a Na-22 point source in the PET image were lower than 1.6 mm in whole the FOV due to the DOI capability. In addition, the influence of the simultaneous measurements on the PET performance is negligible. On the other hand, the SNR of the phantom image in the magnitude images was degraded from 259.7 to 209.4 due to noise contamination from the power supplies.

